# Structural Changes in Molluscan Community over a 15-Year Period before and after the 2011 Great East Japan Earthquake and Subsequent Tsunami around Matsushima Bay, Miyagi Prefecture, Northeastern Japan

**DOI:** 10.1371/journal.pone.0168206

**Published:** 2016-12-09

**Authors:** Shin’ichi Sato, Tomoki Chiba

**Affiliations:** 1 Institute of Geosciences, Shizuoka University, Shizuoka, Japan; 2 Independent researcher, Funabashi City, Chiba Prefecture, Japan; CSIR-National Institute of Oceanography, INDIA

## Abstract

We examined structural changes in the molluscan community for ten years (2001–2010) before and five years (2011–2015) after the 2011 Great East Japan Earthquake and subsequent tsunami around Matsushima Bay, Miyagi Prefecture, northeastern Japan. Before the earthquake and tsunami, *Ruditapes philippinarum*, *Macoma incongrua*, *Pillucina pisidium*, and *Batillaria cumingii* were dominant, and an alien predator *Laguncula pulchella* appeared in 2002 and increased in number. After the tsunami, *R*. *philippinarum* and *M*. *incongrua* populations quickly recovered in 2012, but *P*. *pisidium* and *B*. *cumingii* populations did not recover until 2015. By contrast, *Musculista senhousia*, *Mya arenaria*, *Retusa* sp., and *Solen strictus* were found in low densities before the tsunami, but they rapidly increased in abundance/number over five years after the tsunami. These results suggest that the molluscan community on the Tona Coast was drastically changed by the earthquake and tsunami, and some species mainly inhabiting the intertidal—subtidal zone may have increased in number because of land subsidence. We also emphasize that the seawall delayed recovery of the intertidal community after the earthquake and tsunami.

## Introduction

Benthic animals on tidal flats are frequently damaged by natural and artificial catastrophic events, such as heavy sedimentation [1], storms [2], tsunamis [3–5], hypoxia [6–8], oil spills [9], dredging [10], introduced species [11–14], and reclamation [15], and full recovery from these events can take a minimum of 15–25 years [16]. Therefore, in order to examine the ecological impacts and recovery trajectory from these catastrophic events, long-term datasets from at least 15 years before and after the events are necessary. However, in most cases, pre-event data are scarce or absent, so we cannot correctly demonstrate the influence of such catastrophic events on benthic animals.

**Fig 1:**
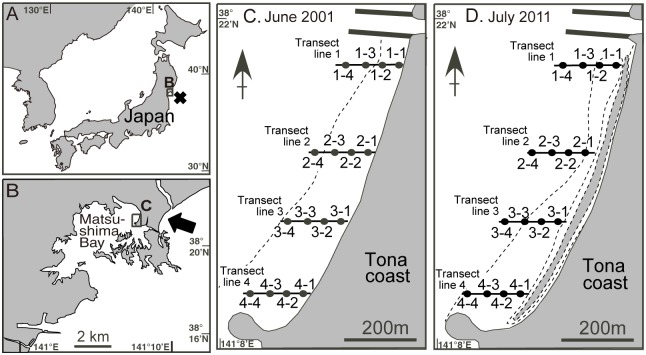
Map of the sampling site around the Tona Coast, Matsushima Bay, Miyagi Prefecture, northeastern Japan [11, 27]. (A) The black box and cross (×) show location of study area and the seismic center of the Great East Japan Earthquake on March 11, 2011, respectively. (B) The black box and the arrow show location of study area and the Nobiru Coast, where the huge tsunami attacked and passed over the land to the Tona Coast, respectively. (C, D) The black circles represent the locations of the 16 sampling stations, with their station numbers, on the Tona Coast, and broken lines mean low water line of spring tides in June 2001 and July 2011, respectively. Gray zone inside of broken line in D indicates deeper area (> 1 m in depth) because of the broken seawalls.

The Great East Japan Earthquake (M 9.0) occurred east of Miyagi Prefecture (Fig 1A) on March 11, 2011, and a huge tsunami devastated a wide area of the Pacific coast from the Tohoku to the Kanto regions [17]. Many researchers reported that the tsunami greatly damaged coastal ecosystems (e.g., [18–26]). However, there were also few long-term datasets of more than 15 years that were collected before and after this tsunami.

We monitored annual changes in species densities of mollusks for ten years before and five years after the 2011 earthquake, and subsequent tsunami, at the Tona Coast (Fig 1B), Higashi-Matsushima City, Miyagi Prefecture [11, 27]. Around the Tona Coast, a huge tsunami arrived from the landside through the Nobiru Coast (arrow in Fig 1B). The height of the tsunami around the Nobiru Coast was 6.93 m [17], and broken seawalls flowed into the Tona Coast [27] (Figs 1D, S1, S2A). Furthermore, the depth of land subsidence that occurred around the sampling area was -0.38 to -0.43 m [28], and most sampling stations on the Tona Coast were no longer exposed even at low tide after the earthquake [27] (Fig 1D). In the present study, we aimed to document the structural changes in the molluscan community over a 15-year period before and after the 2011 Great East Japan Earthquake and subsequent tsunami. With established baseline data before from the region [11, 27], we discuss the recovery trajectory of the molluscan community after this catastrophic event.

## Materials and Methods

### Sample collection

Quadrat sediment samples were collected annually in June or July from 16 fixed stations on the Tona Coast, from 2001 to 2015 (Fig 1). Four transect lines, named from 1 to 4, were established at 250-m intervals along the coast, and four sampling stations were fixed on each transect line at 50-m intervals from land (Fig 1C). Each sampling station was identified using a global positioning system (GPS) receiver (Pockenavi Mini, Empex Ltd.), and samples from one quadrat (25 × 25 cm) were obtained by digging to a depth of 20 cm using a shovel. The samples were sieved with a 2-mm mesh, and living benthic animals were removed from the debris in the laboratory. All living mollusks were identified to species, counted, and preserved in 70% ethanol. The field survey was performed with the permission of the Naruse Branch of the Japan Fisheries Cooperative.

### Statistical analyses

We recorded 4,219 mollusks (24 families, 28 genera and 28 species) from the samples (S1 and S2 Tables). Based on the molluscan literature [29–33], the mollusk species were categorized into “intertidal species” and “intertidal—subtidal species” as they mainly inhabit the intertidal zone and intertidal to subtidal zones, respectively. The number and percent community composition of the mollusk species in each year were shown as bar plots.

All rare species (total abundance < 10) were excluded prior to multivariate analyses because their occurrences in the dataset depend on chance. After excluding rare species, the final data matrix consisted of 4,175 mollusks (ca. 99.0% of all individuals). The final data matrix was divided by the maximum abundance of each species and then divided by total abundance of each year (i.e., Wisconsin double standardization in [34]). Q-mode and R-mode cluster analyses with Ward’s minimum-variance method were used to recognize characteristic species before and after the earthquake and tsunami. For Q-mode and R-mode cluster analyses, the Bray-Curtis dissimilarity and 1-Spearman’s rho were calculated based on the standardized data matrix, respectively. The results of cluster analyses were shown as dendrograms, together with a heatmap of the standardized data matrix.

To quantify the recovery trajectory of the molluscan community after the earthquake and tsunami, we used non-metric multidimensional scaling (nMDS) based on the Bray-Curtis dissimilarity matrix. The species scores were projected onto the nMDS ordination using the weighted averages of the standardized data (following [34]). In the nMDS ordination, year points (2001–2015) that are in close proximity to one another represent similar compositions of the molluscan community, and species points located near a year point indicate the characteristic species of that year. Because the distance between year points in the nMDS ordination represent differences between community compositions, greater distances represent larger changes in the community between years (e.g., [35, 36]). In addition to the R core environment [37], the vegan package [38] was used to analyze the data.

## Results

### Structural changes in molluscan community

**Fig 2:**
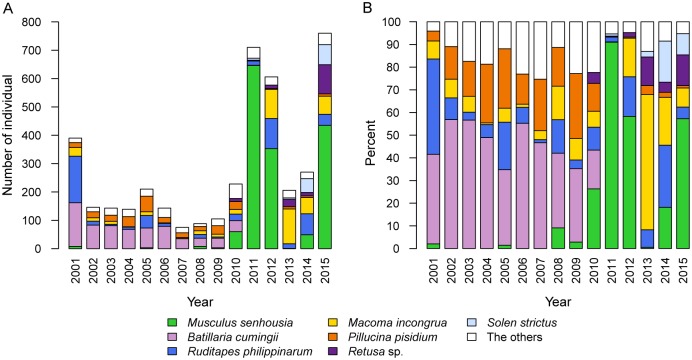
Bar plots showing number (A) and percent community composition (B) of mollusk species on the Tona Coast before (2001–2010) and after (2011–2015) the Great East Japan Earthquake and subsequent tsunami. The species with total abundance < 100 are grouped into “The other” category.

**Fig 3:**
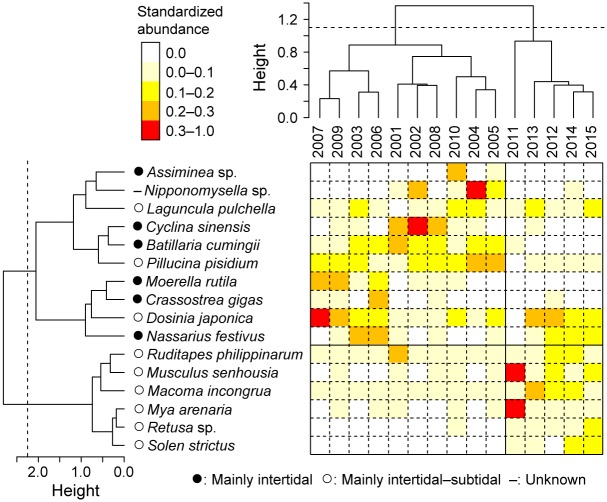
Cluster analyses showing composition of molluscan community on the Tona Coast before (2001–2010) and after (2011–2015) the Great East Japan Earthquake and subsequent tsunami.

**Fig 4:**
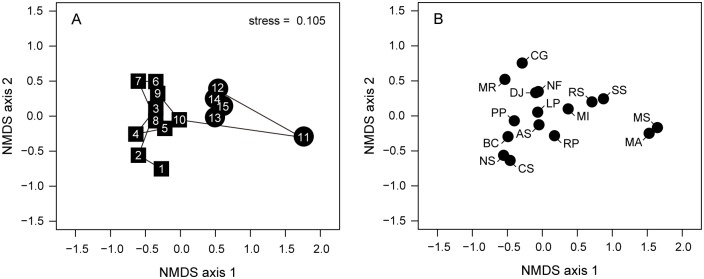
Two-dimensional nMDS (non-metric multidimensional scaling) ordinations highlighting temporal changes in community structure of mollusk species on the Tona Coast. (A) Year scores from the nMDS ordination. The squares and circles represent the years before (2001–2010) and after (2011–2015) the Great East Japan Earthquake and subsequent tsunami, respectively. Lines connect consecutive years (1–15: 2001–2015). (B) Species scores from the nMDS ordination. Species abbreviations: MS *Musculus senhousia*, BC *Batillaria cumingii*, RP *Ruditapes philippinarum*, MI *Macoma incongrua*, PP *Pillucina pisidium*, RS *Retusa* sp., SS *Solen strictus*, LP *Laguncula pulchella*, MA *Mya arenaria*, DJ *Dosinia japonica*, NF *Nassarius festivus*, NS *Nipponomysella* sp., AS *Assiminea* sp., CG *Crassostrea gigas*, CS *Cyclina sinensis*, MR *Moerella rutila*.

The structure of the molluscan community remained relatively stable for the ten years before the 2011 earthquake and tsunami (Figs 2–4). In 2001, *Ruditapes philippinarum*, *Macoma incongrua*, *Pillucina pisidium* and *Batillaria cumingii* were collected in large numbers (Fig 2A), and *R*. *philippinarum* and *B*. *cumingii* occupied more than 80% of the community (Fig 2B). However, an invasive predator *Laguncula pulchella* appeared in 2002 and selectively consumed *R*. *philippinarum* and *M*. *incongrua*, resulting in a decline in these populations between 2002 and 2004 [11, 13, 14]. In contrast, the proportions of *B*. *cumingii* and *P*. *pisidium* increased between 2001 and 2002 (Fig 2B) because these species were less vulnerable to predation by *L*. *pulchella* [11, 12]. The density of these species also decreased between 2005 and 2010 (Fig 2A), because preferred prey species (*R*. *philippinarum* and *M*. *incongrua*) for *L*. *pulchella* became scarce and *L*. *pulchella* began to attack *B*. *cumingii* and *P*. *pisidium* more frequently as suboptimal prey [11, 13, 14].

After the tsunami, all five of the above species decreased in number or disappeared in 2011, but some of them rapidly recovered their populations in 2012 and 2013 (Fig 2). In 2011, only one individual of *B*. *cumingii* and *M*. *incongrua* were collected from the 16 sampling stations, and *P*. *pisidium* were not collected at all (S1 Table). *R*. *philippinarum* was still collected in 2011, and the abundance of *R*. *philippinarum* and *M*. *incongrua* increased in 2012 (Fig 2A). In particular, relative to earlier observations, *M*. *incongrua* was found in much greater densities after the tsunami and occupied more than 50% of the community by 2013 (Fig 2B).

Furthermore, a remarkable component of the community changes was the abrupt increase in *Musculus senhousia* and *Mya arenaria* (Fig 2, S1 and S2 Tables). *M*. *senhousia* was sparse (0.0–26.3%) until 2010, but rapidly increased to 91.0% in 2011 (Fig 2). After this period, the *M*. *senhousia* population fluctuated between 0.5% and 58.3% (Fig 2). *Retusa* sp. and *Solen strictus* also rapidly increased (at most 13.6% and 18.2%, respectively) between 2013 and 2015, although they were rare or absent before the tsunami (Fig 2B).

The results of the cluster analyses show that the molluscan community before the 2001 event was characterized by species that mainly inhabit the intertidal zone (e.g., *B*. *cumingii*, *Assiminea* sp., *Crassostrea gigas*, *Cyclina sinensis*, *Moerella rutila*) (Fig 3). In particular, *B*. *cumingii* was abundant (12.3–56.8%) until 2010, but rarely recorded (0.0–0.1%) thereafter (Figs 2 and S3A). The characteristic elements of the molluscan community after the earthquake and tsunami were species that mainly inhabited the intertidal—subtidal zones and that increased in number (e.g., *R*. *philippinarum*, *M*. *incongrua*, *Retusa* sp.) or first appeared (e.g., *S*. *strictus*) after 2011 (Figs 2, 3 and S3).

### Recovery trajectory of molluscan community

We quantified the relative structural changes in the molluscan community, and shifts in the characteristic species, through time using two-dimensional nMDS ordinations (Fig 4). The largest community change occurred between 2010 and 2011, concurrent with the earthquake and tsunami (Fig 4A). As populations of *R*. *philippinarum* and *M*. *incongrua* increased from 2011 to 2012 (Fig 2), the 2012 data point in the ordination sharply approximates to those of 2001–2010, showing a recovery process from the earthquake and tsunami during this period (Fig 4A). However, the molluscan community remained relatively uniform after 2012 and did not return to its original state (i.e., before 2010) even five years after the earthquake and tsunami (Figs 3 and 4A).

## Discussion

Following the earthquake and tsunami, an abrupt increase in a few species and a persistent decrease in intertidal species caused structural changes in the molluscan community on the Tona Coast (Figs 2–4). Since many benthic organisms were reworked from the sediment and washed away by the tsunami, occasionally transported larvae could colonize the nearly barren habitat without intense competition with the other species. This inference is supported by the observed dominance of small individuals of *M*. *senhousia* and *M*. *arenaria* in 2011 immediately (2 months) after the tsunami on the Tona Coast [27], suggesting that opportunistic species were able to colonize after the tsunami. The decrease in intertidal species can be explained by land subsidence due to the earthquake and coastal erosion by the tsunami, as many of the sampling stations have been unexposed even at low tide since the earthquake [27]. These topographical changes likely prevented settlement and/or survival of the intertidal species, leading to the shift in characteristic species from intertidal to intertidal—subtidal species after the earthquake and the tsunami (Figs 3 and 4).

Before the 2011 earthquake and tsunami, intertidal species such as *B*. *cumingii* were distributed mainly around the uppermost stations in each transect line, but after the event, intertidal—subtidal species became dominant in the same zone (S3 Fig). These results suggest that zonation patterns of these species were moved landward by land subsidence and coastal erosion. *B*. *cumingii* became rare after the earthquake and tsunami, but we observed that many adults of this species were distributed in a patch outside of the quadrat at sampling station 4–1 in July 2011 (S2B Fig) [27]. As *B*. *cumingii* spawns isolated eggs from which juveniles hatch directly [39], it may take longer for this species to increase in abundance than other species with a planktonic larvae stage [27]. Nevertheless, juveniles of *B*. *cumingii* have still not been found inside a quadrat in the five years since the earthquake and tsunami, so it is considered that all sampling stations in this study area are no longer suitable habitat for intertidal species.

There are other potential factors that may influence changes in the molluscan community, such as changed in sediment characteristics, salinity, and algal distribution. Although we have not collected data about these factors, it is important to consider the influences that these factors may have. For example, increases in the deposit-feeding *M*. *incongrua* may reflect changes in sediment characteristics at the study site. As deposit-feeding benthos attain high densities on soft muddy substrates rich in fine organic particles [40, 41], deposition of finer sediment during the post-tsunami period could lead an increase in deposit-feeders, as observed in Paracas Bay, Peru, after a tsunami occurred in 2007 [4]. Salinity is also important for the distribution of mollusk species and increases in intertidal—subtidal species after the earthquake and tsunami may be caused by increased salinity at the study site. Further, changes in food source for mollusk species could be important. After the 2011 event, adjacent area of the study site changed from agricultural fields to wasteland as shown in S1 Fig. These factors may cause changes in the nutritional contents of drainage water, and influence the molluscan community by altering the distribution of algae that are an important food source for mollusk species.

A key consideration in assessing the impact of a huge earthquake and subsequent tsunami on coastal ecosystems is the possibility of synergistic interactions with anthropogenic coastal structures. For example, coastal land uplift following the Maule 2010 earthquake increasing abundance of intertidal species, even in front of existing coastal armoring through restoring intertidal habitat that was once eliminated while developing coastal armoring before the earthquake [5]. In contrast, our results showed that the impacts of a huge earthquake and tsunami on the intertidal community may persist for more than five years in a subsided coastline with seawall, such as the Tona Coast (S2A Fig). It is probable that historically characteristic intertidal species lost their habitat because of land subsidence and coastal erosion, and could not migrate landward since the existing seawall prevented landward extension of the intertidal habitat. We suggest that the seawall, as well as land subsidence, delayed recovery of the intertidal community from the earthquake and the tsunami. After the earthquake, a slow uplift has been observed in the study area [42]. It may be that the intertidal community will recover from the earthquake and tsunami after the slow uplift and sedimentation restore the intertidal habitat that was once lost by rapid co-seismic subsidence and tsunami erosion. As many benthic communities take a minimum of 15–25 years to fully recover from disturbances [16], we need to continue this study for at least another ten years.

## Supporting Information

S1 Table Number of mollusk species collected from the Tona Coast during 2001–2015. (XLSX)

S2 Table Number of mollusk species collected from 16 fixed sampling stations on the Tona Coast during 2001–2015. (XLSX)

S1 Fig Aerial photographs around the Tona Coast before and after the tsunami. (A) April 2002, (B) September 2015. The Google Earth images were obtained in October 2016.
(EPS)

S2 Fig Photographs around the Tona Coast after the tsunami. (A) Southern part of the sampling site (around transect lines 3 and 4) and some broken seawalls (taken by T Chiba in July 2011). (B) *Batillaria cumingii* distributed in a patch outside of the quadrat at the sampling station 4–1 on the Tona Coast [27] (taken by S Sato in July 2011).
(EPS)

S3 Fig Distribution of four dominant species before (2001–2010) and after (2011–2015) the tsunami. (A) *Batillaria cumingii*, (B) *Ruditapes philippinarum*, (C) *Macoma incongrua*, (D) *Musculus senhousia*. Each column indicates density (ind. m^–2^, mean + standard error) per year, but the scale varies among the plots. Original data were reported in [11, 27].
(EPS)
